# Percutaneous coronary intervention for left main coronary artery malperfusion in acute type A aortic dissection

**DOI:** 10.1007/s12928-021-00793-4

**Published:** 2021-07-13

**Authors:** Yuya Taguchi, Shunsuke Kubo, Akihiro Ikuta, Kohei Osakada, Makoto Takamatsu, Kotaro Takahashi, Masanobu Ohya, Takenobu Shimada, Katsuya Miura, Ryosuke Murai, Takeshi Tada, Hiroyuki Tanaka, Yasushi Fuku, Tsuyoshi Goto, Tatsuhiko Komiya, Kazushige Kadota

**Affiliations:** 1 Department of Cardiovascular Medicine, Kurashiki Central Hospital, 1-1-1, Miwa, Kurashiki, 710-8602 Japan; 2 Department of Cardiovascular Surgery, Kurashiki Central Hospital, 1-1-1, Miwa, Kurashiki, Japan

**Keywords:** Percutaneous coronary intervention, Left main coronary artery malperfusion, Acute type A aortic dissection

## Abstract

The clinical outcomes of patients undergoing percutaneous coronary intervention (PCI) for left main coronary artery (LMCA) malperfusion caused by acute type A aortic dissection (AAAD) remains largely unexplored. The aim of this study was to determine the clinical outcomes of patients undergoing PCI for LMCA malperfusion caused by AAAD. We examined nine consecutive patients undergoing PCI for LMCA malperfusion caused by AAAD between 1995 and 2020. The mean age was 55.4 ± 7.7 years. Eight patients presented cardiogenic shock, and five patients cardiopulmonary arrest. Two patients were diagnosed with AAAD before coronary angiography using computed tomography and transthoracic echocardiography, respectively, and in the other seven patients after coronary angiography using other modalities. Four patients underwent PCI on intra-aortic balloon pumping support, and four patients on venoarterial extracorporeal membrane oxygenation (VA-ECMO) support, including one patient on both. PCI was successful in eight patients, with final thrombolysis in myocardial infarction grade 2 or 3. The four patients on VA-ECMO did not undergo aortic dissection repair due to poor recovery of cardiac function and died during the hospital stay, and the other five patients had successful PCI, underwent aortic dissection repair, and remained alive at 5 year follow-up. In conclusion, LMCA malperfusion caused by AAAD seemed to have clinical presentations and electrocardiogram changes similar to acute coronary syndrome. PCI and subsequent surgical aortic repair saved the lives of all AAAD patients with LMCA malperfusion who had not required VA-ECMO.

## Introduction

Aortic dissection is associated with cardiac tamponade, aortic regurgitation, and ischemia in various branches, depending on the progress of dissection. Coronary malperfusion is one of the fatal complications of acute type A aortic dissection (AAAD). The in-hospital mortality rate in AAAD patients without any complications ranges from 8 to 19%, whereas that in AAAD patients with coronary malperfusion ranges from 20 to 30% [1–3]. Malperfusion in the left main coronary artery (LMCA) is particularly associated with catastrophic outcomes, because it can cause cardiogenic shock or cardiac arrest [3, 4]. Immediate revascularization is necessary for AAAD patients with LMCA malperfusion to treat myocardial ischemia and provide hemodynamic stability.

The current guidelines suggest surgical resection and thoracic aorta replacement as the gold standard for the treatment of AAAD [5, 6]. According to previous research, any delay in the door-to-balloon time in patients with ST elevation myocardial infarction (STEMI) undergoing percutaneous coronary intervention (PCI) is associated with higher in-hospital mortality [7], and preoperative PCI is effective for coronary malperfusion caused by AAAD [4, 8–12]. However, regarding LMCA malperfusion in AAAD, its analytical treatment data are scarce because of its low incidence, and, therefore, which treatment should take priority, surgical correction or reperfusion therapy, remains controversial.

Here we present a case series of patients undergoing PCI for LMCA malperfusion caused by AAAD, describe their in-hospital and long-term outcomes, and discuss the optimal treatment strategy.

## Methods

This retrospective, single-center, observational study was based on an initial cohort of 10,530 consecutive patients with acute coronary syndrome undergoing emergent PCI between January 1995 and January 2020. In this cohort, we included patients with LMCA malperfusion caused by AAAD. Patients with right coronary artery malperfusion were not included. The study was done in accordance with the provision of the Declaration of Helsinki and the guidelines for epidemiological studies issued by the Ministry of Health, Labour, and Welfare of Japan. All the patients provided informed consent for the procedure and subsequent data collection, and the patients included in this case series provided consent for publication.

### PCI procedures

When a patient was diagnosed with AAAD after coronary angiography, we immediately referred the patient to cardiovascular surgeons, and emergent surgery was prepared. Then, both reperfusion therapy for the LMCA in the catheterization laboratory to stabilize the hemodynamics and emergent surgery were prepared simultaneously. The final decision was made by the heart team consisting of interventional cardiologists, cardiovascular surgeons, and other medical staff. All PCI procedures were performed in the cardiac catheterization laboratory. Heparin was given to all patients as a bolus intravenously to achieve an activated clotting time of > 250 s. The guidewires were carefully inserted into the left anterior descending artery and left circumflex artery to avoid false lumen insertion. Plain old balloon angioplasty was performed before stenting as needed. Bare metal stents or drug-eluting stents were deployed under angiography or intravascular ultrasound guidance to completely cover the dissection. Intra-aortic balloon pumping (IABP) or a percutaneous left ventricular support device was used in patients with cardiogenic shock, which was defined as systolic blood pressure < 80 mm Hg or the need for parenteral inotropic or vasoactive medication to maintain systolic blood pressure ≥ 80 mm Hg. Venoarterial extracorporeal membrane oxygenation (VA-ECMO) was inserted to patients without return of spontaneous circulation after cardiopulmonary arrest or those with hemodynamic collapse.

### Surgical procedures

Patients were transferred to the operating room after PCI and underwent central repair, except for those with poor cardiac function even after PCI or those with neurological manifestations. After the initiation of extracorporeal circulation and the transection of the ascending aorta, blood cardioplegia was infused from the coronary ostium. In patients with localized aortic dissection at the aortic root, direct repair was performed by closing the false channel with surgical glue. In patients with aortic dissection extending to the ascending aorta, ascending aorta replacement, hemiarch replacement, or total arch replacement was performed. In patients, where complete coronary revascularization was not achieved, coronary artery bypass grafting to the left anterior descending artery and/or the left circumflex artery was performed using saphenous vein grafts.

### Follow-up

We evaluated in-hospital and 5 year outcomes of patients with LMCA malperfusion caused by AAAD by reviewing the hospital charts and contacting the patients.

### Statistical analysis

Data are expressed as mean ± standard deviation for continuous variables. Categorical variables are reported as numbers with relative percentages. SPSS version 25 (International Business Machines, Armonk, NY, USA) was used for all statistical calculations. The data were not analyzed for statistical difference due to the small size of the study population, however.

## Results

### Study population

In the initial cohort, there were 436 patients with culprit lesions in the LMCA, nine of whom with LMCA malperfusion caused by AAAD were reviewed for this case series.

### Patient/clinical characteristics and diagnosis

Baseline patient characteristics of the nine patients are shown in Table 1. No patients had histories of Marfan syndrome, prior aortic dissection or aneurysm, or cardiac surgery. Five of the eight patients with cardiogenic shock had cardiopulmonary arrest due to ventricular fibrillation on arrival, and return of spontaneous circulation was achieved in one patient.

**Table 1:** Baseline patient characteristics

	*n *= 9
Age, yrs	55.4 ± 8.2
Men	8 (89)
Body mass index, kg/m^2^	25.1 ± 5.9
History	
Hypertension	6 (67)
Dyslipidemia	3 (33)
Diabetes	0 (0)
Smoking	6 (67)
Atrial fibrillation	1 (11)
Prior myocardial infarction	1 (11)
Prior cardiac surgery	0 (0)
Patient status	
ST elevation myocardial infarction	7 (78)
Ventricular fibrillation	5 (56)
Cardiogenic shock	8 (89)
Moderate to severe aortic regurgitation	1 (11)
Cardiac tamponade	0 (0)
Laboratory data	
Creatinine, mg/dL	1.13 ± 0.31
Hemoglobin, mg/dL	12.4 ± 3.5

Values are *n* (%) or mean ± standard deviation

**Fig. 1:**
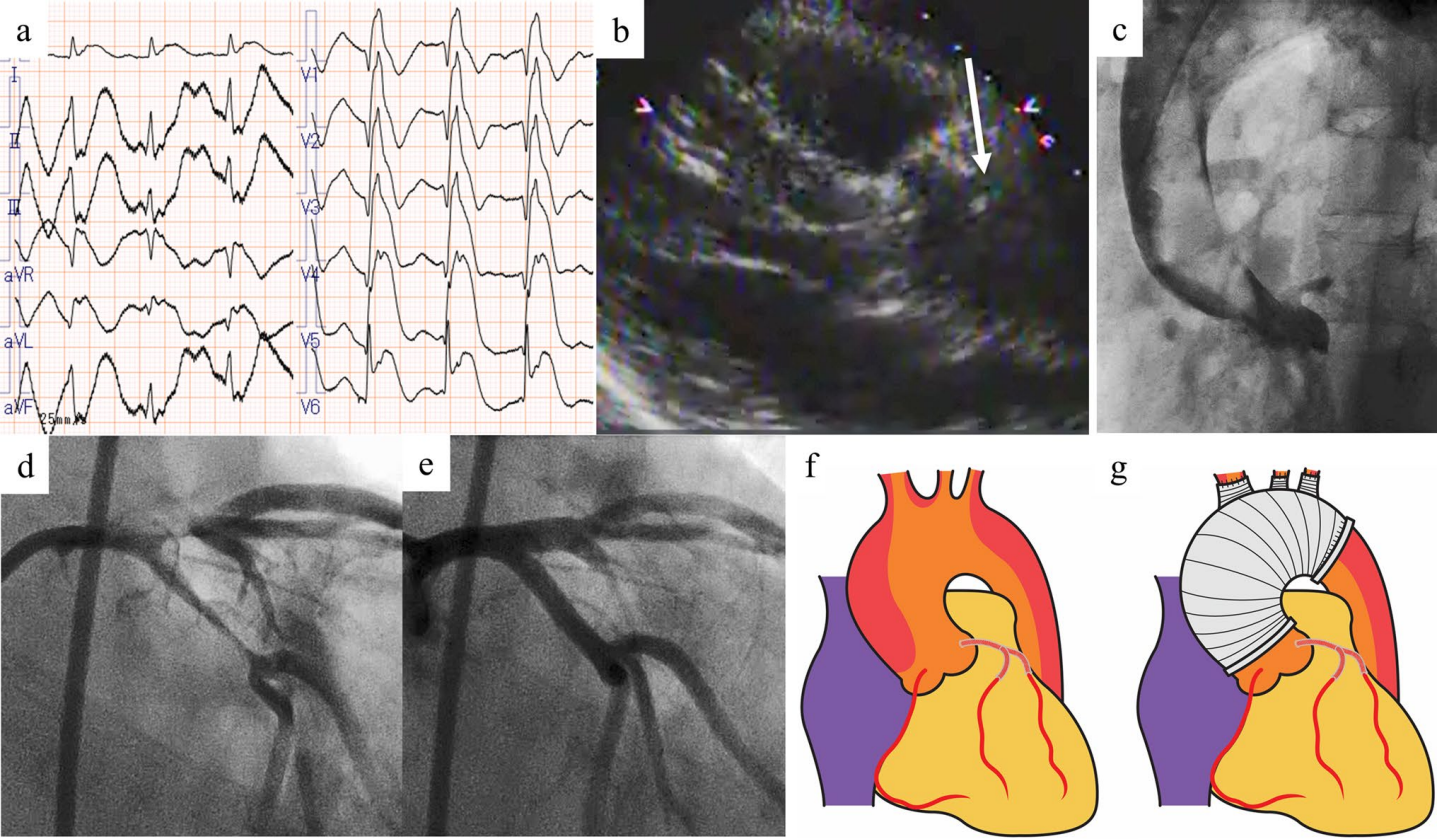
Case diagnosed by transthoracic echocardiography (Case 7). The patient had chest pain, back pain, syncope, and ST segment elevation on electrocardiogram (**a**). ST elevation myocardial infarction was suspected; however, transthoracic echocardiography showed an intimal flap (white arrow) in the ascending aorta, indicating aortic dissection (**b**). A ventricular fibrillation occurred before computed tomography examination, and we immediately transferred him to the cardiac catheterization laboratory for coronary reperfusion. Aortography showed a mobile flap at the ascending to descending aorta (**c**), and coronary angiography showed a true lumen in the left main coronary artery to the left anterior descending and left circumflex arteries compressed by the flap (**d**). Thrombolysis in myocardial infarction (TIMI) 3 flow was achieved by deploying two stents in the left main coronary artery to the left anterior descending and left circumflex arteries (**e**). After percutaneous coronary intervention, total arch replacement was successfully performed [f (before) and **g** (after)]

**Fig. 2:**
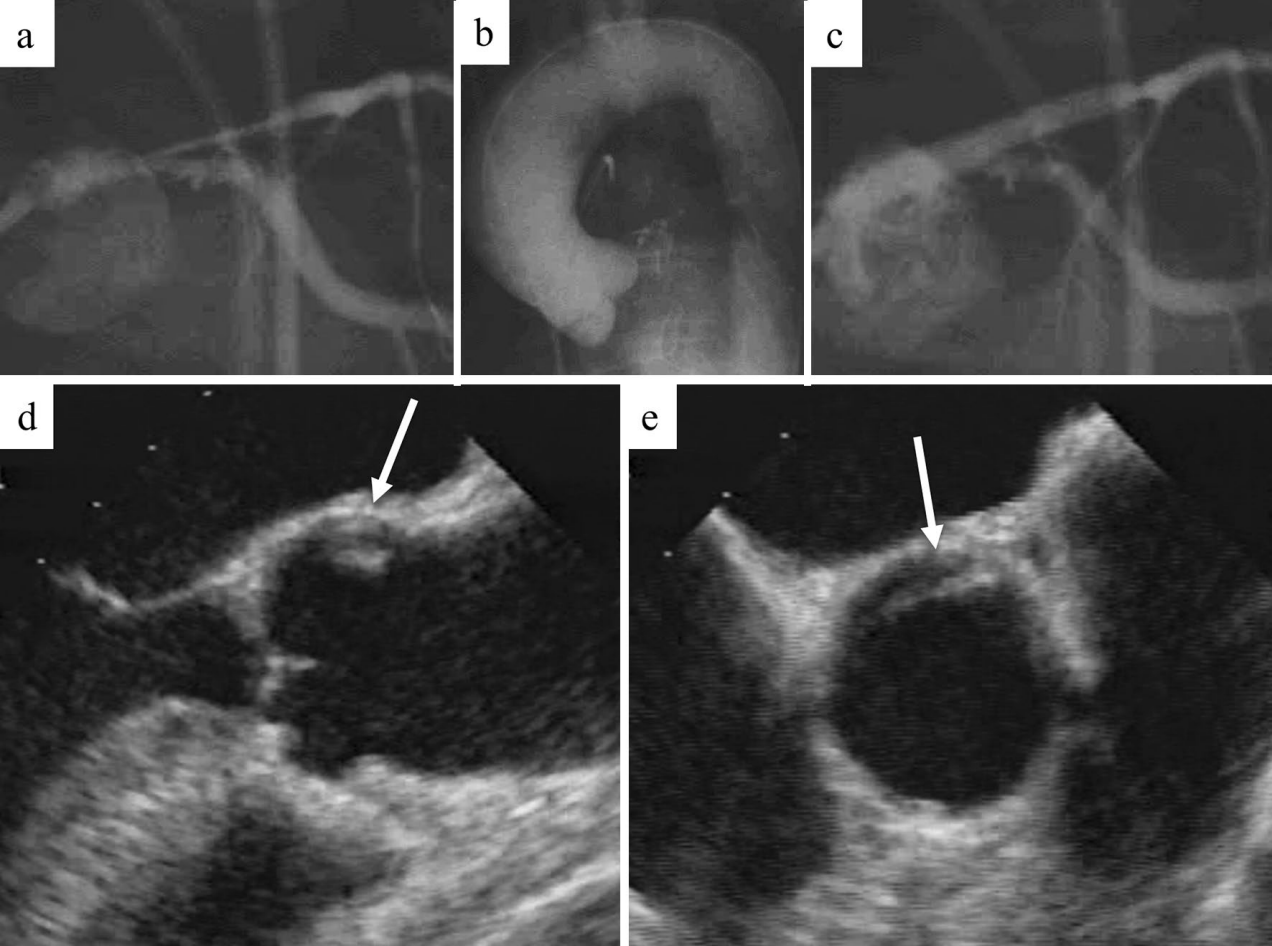
Case diagnosed by transesophageal echocardiography (Case 2). The patient had chest pain and anteroseptal ST elevation myocardial infarction on electrocardiogram. Coronary angiography showed a significant stenosis in the left main coronary artery to the left anterior descending artery compressed by an intimal flap (**a**); however, the flap was not detected by aortography (**b**). Thrombolysis in myocardial infarction (TIMI) 3 flow was obtained by stent implantation in the left main coronary artery to the left anterior descending artery (**c**). After coronary reperfusion, transesophageal echocardiography showed a localized aortic root dissection (white arrow; **d** and **e**)

**Fig. 3:**
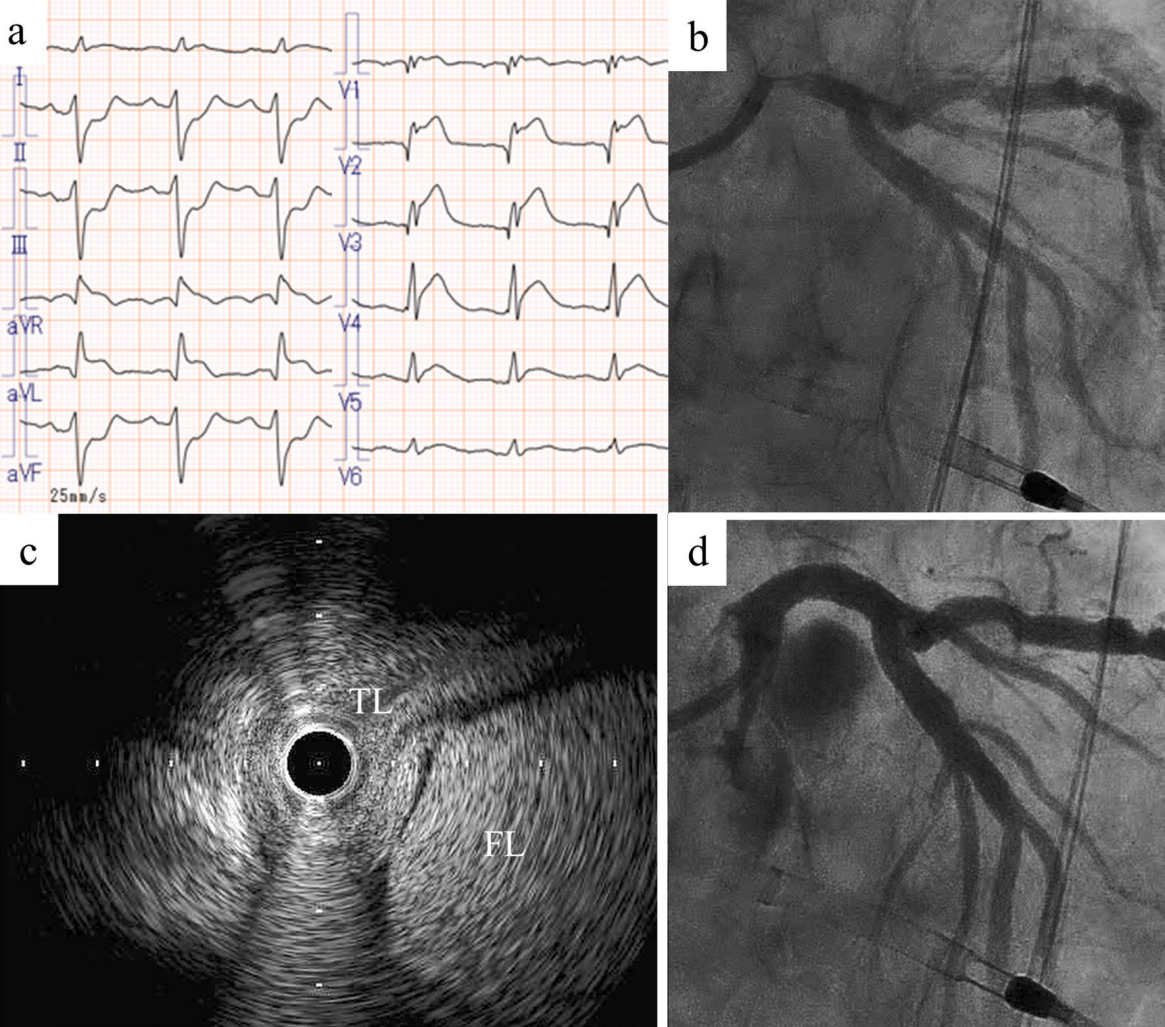
Case diagnosed by intravascular ultrasound (Case 8). The patient had chest pain, bilateral leg pain, and ST-segment elevation on electrocardiogram (**a**). He fell into cardiopulmonary arrest during transportation to the cardiac catheterization laboratory and required venoarterial extracorporeal membrane oxygenation and a percutaneous left ventricular support device. Coronary angiography showed significant stenosis in the left main coronary artery (**b**), and intravascular ultrasound showed a true lumen compressed by a false lumen (**c**). Thrombolysis in myocardial infarction (TIMI) 2 flow was obtained by stent implantation in the left main coronary artery to the left anterior descending artery (**d**). *TL* true lumen, *FL* false lumen

Clinical characteristics and diagnoses are shown in Table 2. Initial presentation was chest pain in seven patients, dyspnea in one patient, and unknown in one patient. The type of acute coronary syndrome was STEMI in seven patient, non-STEMI in one patient, and unknown in one patient. Transthoracic echocardiography showed left ventricular asynergy in all patients and moderate aortic regurgitation in one patient. Only two of the nine patients (22%, Cases 4 and 7) were diagnosed with AAAD before coronary angiography using computed tomography and transthoracic echocardiography, respectively (Fig. 1). The other patients were diagnosed with AAAD after coronary angiography using transesophageal echocardiography, ascending aorta angiography, or intravascular ultrasound (Figs. 2 and 3). Two patients had type A aortic intramural hematoma and the other seven patients type A classic aortic dissection. According to Neri’s classification [1], eight patients had type B and one patient type C. Of the eight patients with Neri’s type B, two patients had LMCA malperfusion caused by false lumen thrombosis.

**Table 2:** Clinical characteristics and diagnoses

Case no	Age	Sex	Chief complaint	Electrocardiogram	TTE	pH	BE (mEq/L)	D-dimer (μg/mL)	Diagnosis before CAG	Diagnostic modality
1	52	M	NA	NA	Asynergy at anteroseptal/posterolateral	NA	NA	NA	NA	NA
					Mild aortic regurgitation					
					EF 35%					
2	49	F	Chest pain	ST elevation in I, aVL, V1–V4	Asynergy at anteroseptal	7.359	+ 1.7	0.6	−	TEE
					Mild aortic regurgitation					
					EF 40%					
3	46	M	Chest pain	ST elevation in I, aVL, V3–V6	Asynergy at anteroseptal	7.246	− 10.0	47	−	AoG
					Small pericardial effusion					
					EF 20%					
4	49	M	Chest pain	ST elevation in I, aVL, V2–V6	Asynergy at anterolateral	7.357	− 2.0	2.8	+	CT
					EF 25%					
5	51	M	Chest pain	ST depression in V2–V6	Asynergy at anterior	7.469	− 4.1	0.8	−	AoG
					EF 30%					
6	58	M	Chest pain	ST elevation in global induction	Asynergy at anteroseptal/posterolateral	NA	NA	NA	−	AoG
					Mild aortic regurgitation					
					EF 15%					
7	67	M	Chest and back pain	ST elevation in I, aVR, aVL, V2–V6	Asynergy at anteroseptal	7.379	− 3.3	6.6	+	TTE
					Moderate aortic regurgitation					
			Syncope		EF 40%					
					Flap					
8	69	M	Chest pain	ST elevation in I, aVR, aVL, V1–V6	Asynergy at anteroseptal/posterolateral	7.385	− 11.6	671	−	IVUS
			Bilateral leg pain		Small pericardial effusion					
					EF 30%					
9	58	M	Dyspnea	ST elevation in I, aVR, aVL, V1–V5	Asynergy at anteroseptal/posterolateral	7.028	− 20.4	346	−	AoG
					EF 20%					

*AoG* aortography, *BE* base excess, *CAG* coronary angiography, *CT* computed tomography, *EF* ejection fraction, *IVUS* intravascular ultrasound, *NA* not available, *TEE* transesophageal echocardiography, *TTE* transthoracic echocardiography

### Procedures

**Fig. 4:**
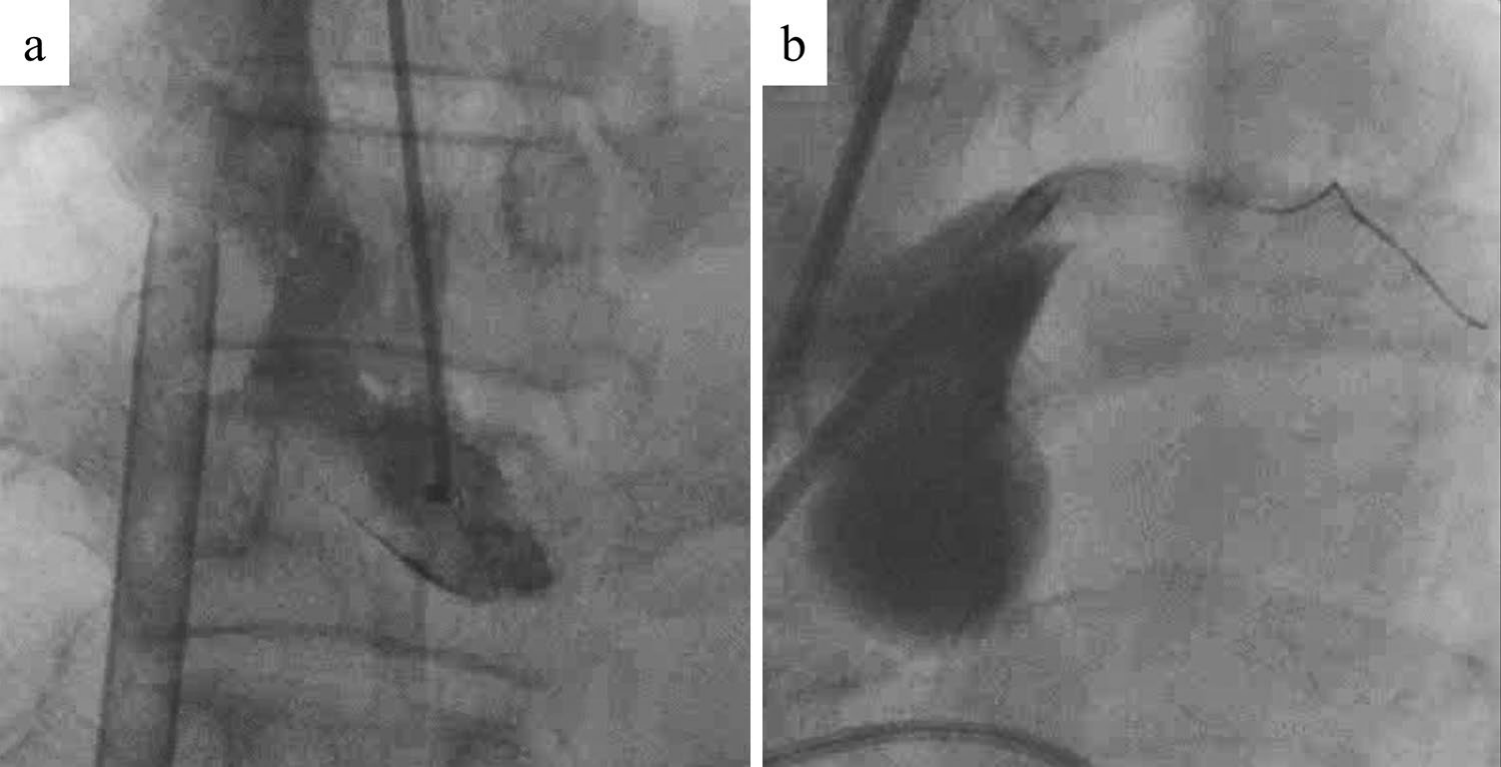
Case where percutaneous coronary intervention was unsuccessful (Case 6). The patient had chest pain and ST segment elevation on electrocardiogram. He fell into cardiopulmonary arrest during transportation to the cardiac catheterization laboratory and required venoarterial extracorporeal membrane oxygenation. Aortography showed a mobile flap at the ascending to descending aorta, and coronary angiography showed total occlusion of both the left main coronary artery and right coronary artery (**a**). Percutaneous coronary intervention was performed but unsuccessful, because the guidewire failed to pass through the true lumen (**b**)

The procedures are shown in Table 3. Among the eight patients with cardiogenic shock, four required IABP and one a percutaneous left ventricular support device. In all patients, VA-ECMO was used in four patients (44%) requiring persistent ventricular fibrillation, coronary reperfusion was achieved in eight patients (89%), except for one patient, where the guidewire failed to pass through the true lumen (Fig. 4), and stents were implanted in seven patients (78%). Final kissing balloon inflation after LMCA crossover stenting was performed in two patients. Perfusion catheter insertion after plain old balloon angioplasty was performed in only one patient. The final thrombolysis in myocardial infarction (TIMI) score was 3 in four patients, 2 in four patients, and 0 in one patient. Among eight patients who achieved coronary reperfusion, five were immediately transferred to the operating room to undergo central repair, two of whom were treated by direct repair with false lumen closure and the other three were treated by ascending aorta replacement, hemiarch replacement, and total arch replacement, respectively. In three patients, where complete coronary revascularization was not achieved by PCI, coronary artery bypass grafting using saphenous vein grafts was additionally performed. The other four patients (44%) did not undergo surgery because of insufficient cardiac function and VA-ECMO dependence.

**Table 3:** Procedures and outcomes

Case no	Cardiogenic shock	Systolic blood pressure (mmHg)	CPA	MCS	System	CAG	Neri type	Percutaneous coronary intervention	Onset to balloon time (min)	Final TIMI flow	Peak CK/CK-MB (IU/L)	Surgery	EF at discharge (%)	Outcome
1	+	NA	−	IABP	Femoral.A	#5 99%	B	LMT-LAD stenting (BMS × 2)	NA	3	NA	Ascending aorta replacement	NA	Alive
					7Fr JL 4.0 ST	#6 99%						CABG (SVG–LAD, SVG–LCx)		
2	−	124	−	–	Femoral.A	#5 99%	B	LMT-LAD stenting (BMS × 3)	183	3	7017/273	Closure of the false channel	49	Alive
					7Fr JL 3.5 ST	#6 99%		KBT				CABG (SVG–LCx)		
3	+	64	+	VA-ECMO	Femoral.A	#5 99%	B	LMT stenting (BMS)	278	3	13,901/300 <	–	–	Dead
				IABP	7Fr JL 4.0 ST									
4	+	75	−	IABP	Femoral.A	#5 100%	B	POBA + perfusion catheter for LAD and LCx	250	2	9908/203	Closure of the false channel	29	Alive
					8Fr JL 4.0							CABG (SVG–LAD, SVG–LCx)		
5	+	65	−	IABP	Femoral.A	#5 99%	B	LMT stenting (BMS)	153	2	3282/230	Hemiarch replacement	49	Alive
					7Fr JL 5.0									
6	+	52	+	VA-ECMO	Femoral.A	#1 100%	C	Failure (guidewire failure)	–	0	NA	–	–	Dead
					7Fr AL 1.0	#5 100%								
7	+	80	+	–	Femoral.A	#5 99%	B	LMT–LAD stenting (BMS)	224	3	4951/213	Total arch replacement	48	Alive
					7Fr AL 1.0	#6 99%		LMT–LCx stenting (BMS)						
						#11 99%		KBT						
8	+	71	+	VA-ECMO	Femoral.A	#5 99%	B	LMT stenting (DES)	406	2	NA	–	–	Dead
				Impella	7Fr SL 4.0									
9	+	90	+	VA-ECMO	Brachial.A	#5 99%	B	LMT–LAD stenting (DES)	206	2	NA	–	–	Dead
					7Fr SL 4.0	#6 99%								

*BMS* bare metal stent, *CABG* coronary artery bypass grafting, *CK* creatine kinase, *CK-MB* creatine kinase myocardial band, *CPA* cardiopulmonary arrest, *DES* drug-eluting stent, *EF* ejection fraction, *IABP* intra-aortic balloon pumping, *KBT* kissing balloon technique, *LMT* left main trunk, *LAD* left anterior descending coronary artery, *LCx* left circumflex coronary artery, *MCS* mechanical circulatory support, *PCI* percutaneous coronary intervention, *POBA* plain old balloon angioplasty, *SVG* saphenous vein graft; *VA-ECMO* venoarterial extracorporeal membrane oxygenation

### Outcomes

The outcomes are shown in Table 3. Four patients (44%) died in hospital, without VA-ECMO weaning. The other five patients (56%) survived to discharge after successful PCI and surgery, one of whom developed cerebral infarction after surgery. Between survivors and non-survivors, non-survivors had higher D-dimer levels (354.7 vs. 2.7 μg/mL) and more severe metabolic acidosis (pH 7.220 ± 0.180 vs. 7.391 ± 0.053; base excess − 14.0 ± 5.6 vs. − 1.9 ± 2.6 mEq/L). Among the five survivors, two received the diagnosis of AAAD before coronary angiography and three were supported by IABP alone. The mean left ventricular ejection fraction at discharge was 44 ± 10%. All five survivors were alive at 5 year follow-up, one of whom underwent thoracic endovascular aortic repair for aortic aneurysm due to residual dissection (Case 7). No survivors required either hospitalization for heart failure or target vessel revascularization during the follow-up period.

## Discussion

This case series is the first report to systematically investigate the clinical characteristics, procedures, and outcomes in patients undergoing PCI for LMCA malperfusion caused by AAAD. The main findings of this study were as follows: first, AAAD was difficult to diagnose accurately in the emergency room; second, the in-hospital mortality rate in AAAD patients with LMCA malperfusion undergoing PCI was 44%; and third, patients with poor prognosis (non-survivors) tended to have cardiopulmonary arrest requiring VA-ECMO, higher D-dimer levels, and severe metabolic acidosis.

Clinical presentation of AAAD is similar to that of acute coronary syndrome, which frequently results in misdiagnosis [13]. In this study, seven (78%) of the nine patients were diagnosed with acute coronary syndrome in the emergency room and were transferred to the cardiac catheterization laboratory. All patients underwent bedside transthoracic echocardiography; however, a mobile flap was found in the ascending aorta of only one patient (Case 7). According to previous research, three important findings of transthoracic echocardiography indicating AAAD are aortic root dilation, aortic eccentric regurgitation, and pericardial effusion [14]. Because both two patients who were diagnosed with AAAD before coronary angiography survived to discharge, early diagnosis of AAAD may contribute to raise the survival rate. However, diagnosing AAAD in patients with hemodynamic instability is difficult in emergency situations. In this study, although seven of the nine patients were diagnosed with AAAD after coronary angiography, reperfusion therapy in the LMCA was performed without any delay. Because hemodynamic stabilization has to be the top priority, the initial treatment strategy for this condition is similar to that for acute coronary syndrome with cardiogenic shock. In patients with right coronary artery malperfusion who have less hemodynamic instability and lower mortality, surgical aortic repair should be selected as an initial treatment strategy [3]. If AAAD is diagnosed prior to coronary angiography, the patient may benefit from being transported to a hybrid operating room immediately and undergoing reperfusion therapy for the LMCA and subsequent surgical aortic repair.

In this study, both the in-hospital and 5 year follow-up survival rates were 56% (5/9 patients), without either hospitalization for heart failure or target vessel revascularization, which were better than those of previous studies on patients with acute myocardial infarction due to LMCA disease (in-hospital: approximately 50%; 6 month follow-up: 41%) [15–17]. Comorbid aortic dissection may not affect the in-hospital and long-term outcomes, and early reperfusion therapy may contribute to preserve left ventricular function and prevent heart failure. In addition, the etiology of myocardial ischemia in our study population was not atherosclerotic disease, which may have led to the favorable long-term outcomes.

The presence of myocardial ischemia, cardiopulmonary arrest on arrival, and LMCA ischemia have been reported to be risk factors for operative death and postoperative low cardiac output syndrome in AAAD patients [3]. In this study, eight (89%) of the nine patients had cardiogenic shock, which indicates that our study population suffered severe hemodynamic instability. Fatal myocardial ischemia tends to occur before surgical reconstruction of coronary blood flow. According to previous research, the shorter door-to-balloon time decreases the mortality rate in STEMI patients by reducing myocardial damages [7, 18, 19]. In this study, because all patients with severe metabolic acidosis and hemodynamic deterioration requiring VA-ECMO could not be saved, preoperative PCI may be an effective approach to reduce myocardial damages and stabilize hemodynamics in patients with LMCA malperfusion caused by AAAD. IABP as circulatory support is considered a contraindication for AAAD patients because of the concerns over flap extension and aortic rupture. In this study, the four patients were supported by IABP to maintain hemodynamic stability and had no adverse events associated with IABP, and the three patients supported by IABP alone were successfully treated by PCI and subsequent surgery and survived to discharge. Using IABP may be one of the strategies to prevent prolonged cardiogenic shock and subsequent progressive acidosis.

The current study has several limitations. First, this is a retrospective, single-center, observational study with a small study population. Larger prospective studies are warranted to confirm the findings of this study. Second, surgical strategies for AAAD patients with LMCA malperfusion were not assessed. Third, AAAD patients with LMCA malperfusion who had been directly transferred from the emergency room to the operating room were not included. Fourth, the differences in the clinical characteristics between AAAD patients with LMCA malperfusion and acute coronary syndrome patients with LMCA atherosclerosis were not evaluated in this case series. Finally, accurate drug information on perioperative antithrombotic therapy could not be collected.

## Conclusions

LMCA malperfusion caused by AAAD seemed to have clinical presentations and electrocardiogram changes similar to acute coronary syndrome. PCI and subsequent surgical aortic repair saved the lives of all AAAD patients with LMCA malperfusion who had not required VA-ECMO.
